# A quantitative trait locus for nictation behavior on chromosome V

**DOI:** 10.17912/W23D39

**Published:** 2017-09-19

**Authors:** Jun Kim, Daehan Lee, Junho Lee

**Affiliations:** 1 Department of Biological Sciences, Institute of Molecular Biology and Genetics, Seoul National University, Seoul 08826, Korea; 2 Department of Molecular Biosciences, Northwestern University, Evanston, IL, 60208, USA

**Figure 1.**
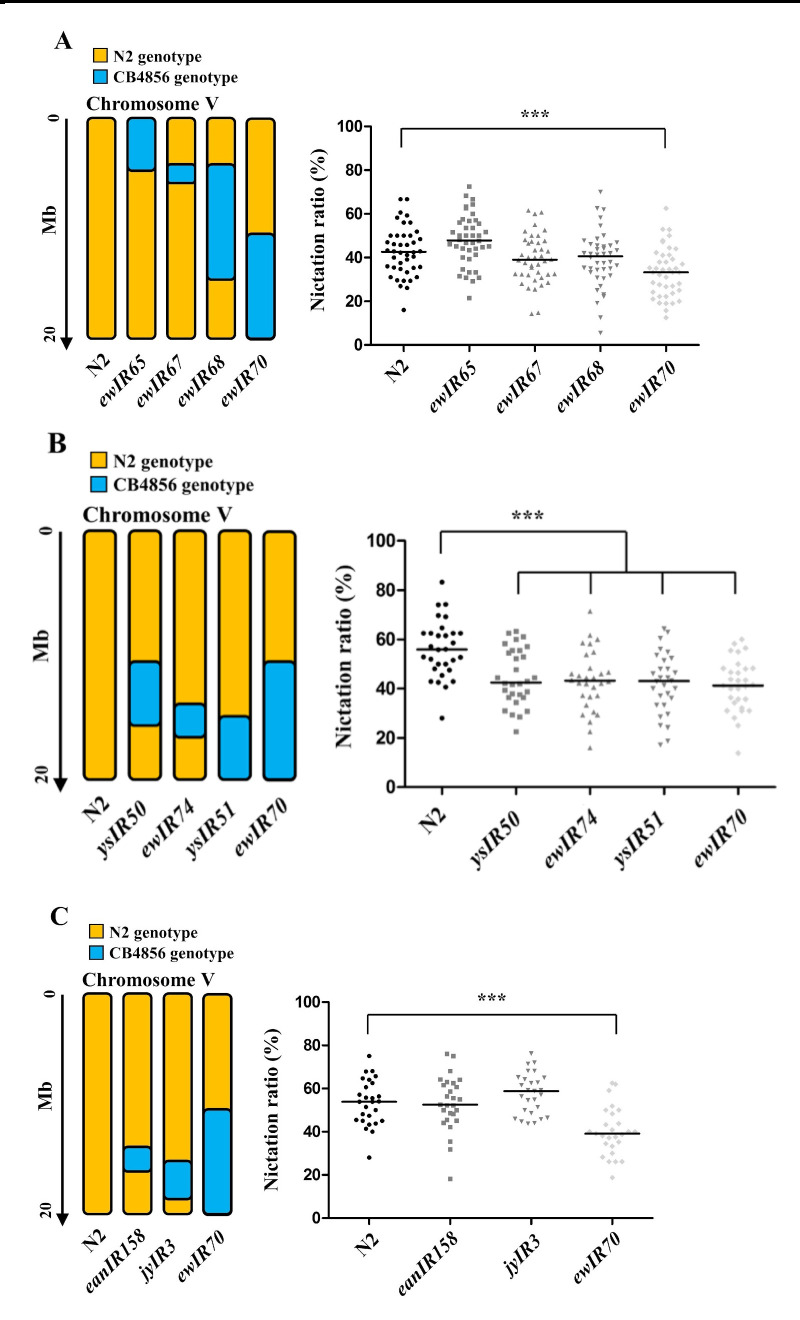
Nictation ratio of N2 x CB4856 introgression lines on chromosome V. Note that the genotypes of some of these introgression lines including *ysIR50* and *ysIR51* are estimates from polymorphism markers, not from whole-genome sequencing. Statistics were analyzed by GraphPad Prism and its One-way ANOVA and Dunnett post-test. All tested groups passed the D’Agostino and Pearson omnibus normality test. Each bar indicates the median of each group. *** P<0.001, significantly different from the control strain, N2.

## Description

Lee et al., 2017 showed that CB4856 has a lower nictation ratio (the number of nictating dauers over the number of moving dauers on a micro-dirt chip) than N2, and that a genetic locus (*nict-1*) on chromosome IV mediates this phenotype difference. Although this paper had no evidence of any other genetic loci for nictation behavior, we have studied other quantitative trait loci (QTL) using different linkage-based mapping strategies. Here, we report another genetic locus that was identified using introgression lines on chromosome V.

N2 x CB4856 introgression lines were tested using a population nictation assay (Lee et al., 2015). We measured the nictation ratios of the *ewIR65*, *ewIR67*, *ewIR6*8, and *ewIR70* lines, which contain N2-type genome in all chromosomes except for CB4856-type chromosome V segments (Figure 1A). Among them, only *ewIR70* line showed significant lower nictation ratio. Therefore, we narrowed down the chromosome V right region.

We also measured nictation ratios of *ysIR50*, *ewIR74*, and *ysIR51* lines (Figure 1B). *ysIR50* and *ysIR51* were derived from N2 x *ewIR70*. *ysIR50*, *ewIR74*, and *ysIR51* lines showed significantly lower nictation ratios than that of N2. Because the putatively responsible QTL region is covered by another near-isogenic region, *eanIR158*, which was made independently by Erik Andersen’s laboratory, we measured the nictation ratios of *eanIR158* and *jyIR3* lines and found that they did not show any significant difference from N2 (Figure 1C).

*eanIR158* and *jyIR3* lines were built with QX1430 x N2 (personal communication) and QX217 x N2 (Balla et al., 2015), respectively, and QX1430 and QX217 were made by Leonid Kruglyak’s laboratory (Andersen et al., 2015; Rockmen and Kruglyak, 2009). However, *ewIR70* and *ewIR74* were built by Jan Kammenga’s laboratory (Doroszuk et al., 2009). Thus, we concluded that the two laboratories may have used different N2 or CB4856strain derivatives, which may have altered genetic variations in the introgression lines. A take-home lesson is that it is important to examine NILs derived from common original strains in order to follow QTL without being affected by differences in background genotypes.

Jan Kammenga kindly provided their N2 x CB4856 introgression lines for chromosome V, which include *ewIR65*, *ewIR67*, *ewIR68*, *ewIR70*, and *ewIR74*, and Erik Andersen also gave us many introgression lines, which include *eanIR158* and *jyIR3* covering 15-19 Mb of chromosome V. We appreciate their help.

## Reagents

Strains: N2, LJ1250 *ysIR50 (V,* *CB4856**>**N2**)*, LJ1251 *ysIR51 (V,* *CB4856**>**N2**)*, ECA238 *eanIR158 (V,* *CB4856**,* *N2**)*, and ERT248 *jyIR3 (V,* *CB4856**>**N2**)*.

Genotypes: *ewIR65 (V,* *CB4856**>**N2**)*, *ewIR67 (V,* *CB4856**>*N2*)*, *ewIR68 (V,* *CB4856**>**N2**)*, *ewIR70 (V,* *CB4856**>**N2**)*, and *ewIR74 (V,* *CB4856**>**N2**)*.
